# Efficacy, Chemical Constituents, and Pharmacological Actions of *Radix Paeoniae Rubra* and *Radix Paeoniae Alba*

**DOI:** 10.3389/fphar.2020.01054

**Published:** 2020-07-10

**Authors:** Yu-Qing Tan, Heng-Wen Chen, Jun Li, Qing-Juan Wu

**Affiliations:** ^1^ Department of Cardiology, Guang’anmen Hospital, China Academy of Chinese Medical Sciences, Beijing, China; ^2^ Graduate School of Beijing University of Chinese Medicine, Beijing University of Chinese Medicine, Beijing, China

**Keywords:** *Radix Paeoniae Rubra*, *Radix Paeoniae Alba*, pharmacological actions, efficacy, chemical constituents

## Abstract

*Radix Paeoniae Rubra* and *Radix Paeoniae Alba* are the different characteristic forms of *Paeonia lactiflora* Pall. They are widely used as traditional Chinese medicines in clinical practices. This study analyzes the development history, efficacy, chemical compositions, and pharmacological effects of *Radix Paeoniae Rubra* and *Radix Paeoniae Alba*, and explores the causes of the similarities and differences of these two amalgams. It provides a basis for the clinical application of these two Chinese medicinal materials, and lays a foundation for further study of the pharmacological effects and the quality identification of *Paeonia lactiflora* Pall as it applies to traditional Chinese medicine.

## Introduction

### The Medical History of *Paeonia lactiflora* Pall

*Paeonia lactiflora* Pall. (PLP, also known as *shaoyao*) is a plant of the genus Paeonia, which is divided into *Radix Paeoniae Rubra* (RPR, also known as *chishao* or red peony root) and *Radix Paeoniae Alba* (RPA, also known as *baishao* or white peony root). As it is a commonly used traditional Chinese medicine even in modern times, PLP has a long history of applications, and it was first applied to medicine as described in the *Prescriptions for Fifty-two Aliments* (Yan, 2005), which can be traced back to the Spring and Autumn Period (771–476 BC). Shen Nong’s *Classic of Medicinal Herbs* (Ma, 1995), the earliest extant monograph belonging to the category of traditional Chinese pharmacology, was compiled approximately during the Qin and Han dynasty (221 BC–220 AD), and it is also the first monograph that describes many pharmaceutical theories, such as the four natures and five flavors of PLP. The *Treatise on Cold-Induced and Miscellaneous Diseases* (Zhang et al., 2019) was written by Zhang Zhongjing at the end of East Han dynasty (25–220 AD). This outstanding work is well known as “the ancestor of formula” and one of the four classics of traditional Chinese medicine. There is still no clear differentiation between RPR and RPA in these works, even though PLP has a wide range of applications by extrapolation from the high frequency of its mention in this work. The *Variorum of Classic of Materia Medica* (480–498 AD) (Tao, 1955) explicitly proposes the viewpoint that PLP has the characteristics of these two mixtures. Unfortunately, the differences in medicinal properties, efficacy, and indicated usage of RPR and RPA provide no additional clarification of this. During the Sui and Tang dynasties (581–906 AD), the first official Chinese pharmacopoeia was released, titled the *Newly Compiled Materia Medica of the Tang Dynasty* (AD 659) (Su, 2004). In this book, RPR and RPA are loosely both defined as PLP. At the same time, a small number of doctors distinguish RPR and RPA in clinical practice. According to medical texts, the mainstream appellation is still based on PLP. Only in the Northern Song Dynasty (960–1127 AD), according to the records in *Kai Bao Materia Medica* (973–934 AD) (Lu and Li, 1998) and *The Peaceful Holy Benevolence Formulae* (978–992 AD) (Hu and Guo, 1996), did traditional Chinese medicine physicians start to distinguish the efficacy of RPA versus RPR and draw a distinction between these two Chinese herbs. The differences and various applications of these were vastly impacted in later generations in the Song, Jin, and Yuan Dynasties (960–1368 AD). In the Ming and Qing Dynasties (1368–1912 AD), the *Materia Medica of South Yunnan* and *Introduction to Medicine* (Cai et al., 1997) describe RPR and RPA in respective terms as two different compounds. These two later works not only clearly distinguish the flavors and natures of RPR and RPA as medicinals and delineate their efficacy, but they also describe their meridian entries and processing as medicinals. Physicians of later generations have made specific supplements from these and have further developed their use based on their clinical experience. The medical history in thought regarding *Paeonia lactiflora* Pall. are shown in **Table 1**.

**Table 1:** The medical history of *Paeonia lactiflora* Pall.

Dynasty	Representative works	Distinction
The spring and autumn periods (771–476 BC)	Prescriptions for 52 Aliments (Yan, 2005)	Earliest recorded
Qin and Han dynasty (221 BC–220 AD)	Shen Nong’s Classic of Medicinal Herbs (Ma, 1995)	Describes pharmaceutical theories, such as four natures and five flavors
	Treatise on Cold-Induced and Miscellaneous Diseases (Zhang et al., 2019)	No clear differentiation between RPR and RPA
Northern and Southern dynasties (420–581AD)	Variorum of Classic of Materia Medica (Tao, 1955)	Refers to the different species of *Paeonia lactiflora* Pall.
Sui and Tang dynasties (581–906 AD)	Newly Compiled Materia Medica of the Tang Dynasty (Su, 2004)	The mainstream appellation is still based on *Paeonia lactiflora* Pall.
Song, Jin, and Yuan dynasties (960–1368 AD)	Kai Bao Materia Medica (Lu and Li, 1998), The Peaceful Holy Benevolence Formulae (Hu and Guo, 1996)	Distinguishes efficacy and indication between *Radix Paeoniae Rubra* and *Radix Paeoniae Alba*
Ming and Qing dynasties (1368–1912 AD)	Materia Medica of South Yunnan, Introduction to Medicine (Cai et al., 1997)	Divides RPR and RPA into two separate drugs

Radix Paeoniae Rubra (RPR), Radix Paeoniae Alba (RPA).

### Sources of *Radix Paeoniae Rubra* and *Radix Paeoniae Alba*

**Figure 1:**
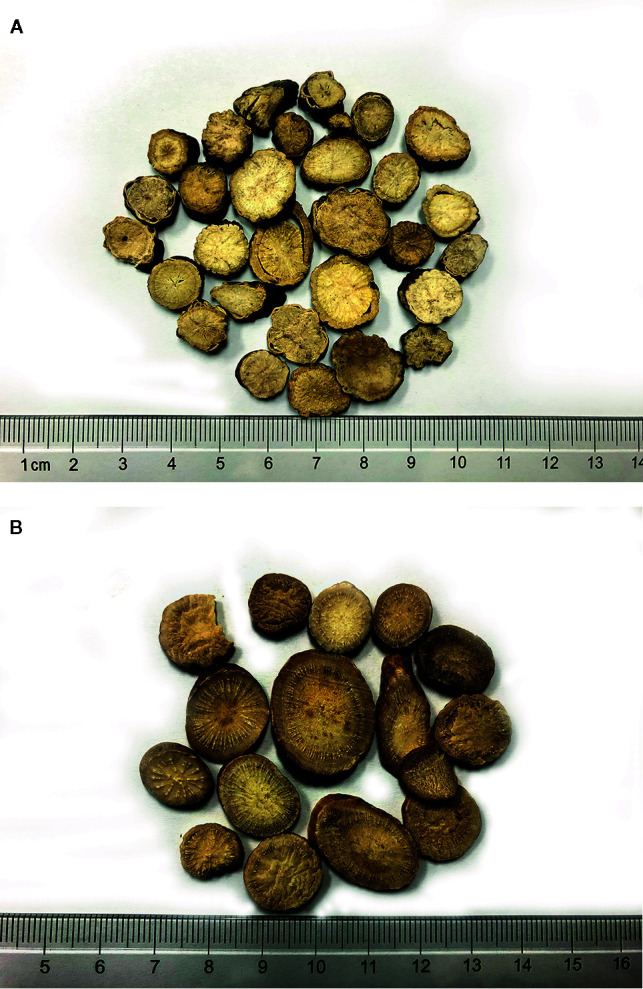
**(A)** Characteristics of RPR. **(B)** Characteristics of RPA.

RPR and RPA are included in the *Chinese Pharmacopoeia* as two different traditional Chinese medicines, but they have many common features in terms of their biological basis. From the perspective of plant origin and biology, both RPR and RPA are derived from the radix of Paeonia belonging to ranunculaceae. RPR is the dried root of PLP, most often found in the wild. The crude herb can be used as a medicine, and is mainly produced in Inner Mongolia, Heilongjiang, Liaoning, and Sichuan. RPA is the dried root of PLP, but the processing method of RPA requires boiling and peeling, in either order, and then drying. RPA is mainly produced in Anhui, Zhejiang, and Hangzhou, most often from cultivated PLP. Nowadays, RPR and RPA can be distinguished according to the following aspects: their processing method, wild or cultivated origin, different production areas, and the chemical content of paeoniflorin (Chinese Pharmacopoeia Commission, 2015). The characteristics of medicinal slices are different between RPR and RPA (**Figures 1A, B** **)**, and can also be identified by microscopic identification, thin layer chromatography, HPLC content determination, infrared chromatography, and UPLC fingerprint. According to *Chinese Pharmacopoeia*, the paeoniflorin (C_23_H_28_O_11_) in RPR is not less than 1.8%, and the paeoniflorin (C_23_H_28_O_11_) in RPA is not less than 1.6% (Chinese Pharmacopoeia Commission, 2015).

*Radix Paeoniae Rubra* (RPR): Rhizome drying directly, the surface is tan, rough, and some integument is easily to peel off. It is hard and brittle, easy to break, with narrow skin. The radial texture of the wood is obvious, some with clefts. *Radix Paeoniae Alba* (RPA): Removing the skin after boiling in boiling water or boiling after removing the skin and drying. The surface is off-white or light brownish red, smooth. Texture compact, not easy to break, cambium ring distinct, rays radiate.

### The Similarities and Differences in the Efficacy of RPR and RPA

Both RPR and RPA are commonly used in traditional Chinese medicine, and while the two herbs are widely applied in the clinical practice, they have different benefits. Yin and Yang are the extensions of ancient Chinese philosophical thoughts. They are the generalization of the opposing attributes of certain things in nature, such as sun and moon, day and night, water and fire. Traditional Chinese medicine (TCM) believes that yin and yang can be transformed into each other, and the essence of the disease is that yin and yang are out of balance. Liver yin is relative to liver yang. Liver yin has the function of nourishing and moisturizing, which can restrict the excess of liver yang. The balance of yin and yang can maintain the normal physiological function of the liver. The flavors and natures of RPR and RPA are slightly cold and enter the liver meridian (He et al., 2012). RPR belongs to the heat-clearing blood-cooling medicinal category, is slightly cold in property, bitter in flavor, and has the function of clearing heat and cooling the blood, as well as dissipating stasis to relieve pain. RPA belongs to the blood-tonifying medicinal category, is also slightly cold in property, bitter, and sour in flavor, and has the functions of nourishing the blood and regulating menstruation, arresting yin to check sweating, emolliating the liver and relieving pain, calming and repressing liver yang (Li et al., 2009; Gao, 2012). These characteristics all play a significant role in treating blood diseases (He et al., 2012). RPR is mainly used for the treatment of heat entering blood constrictions, macula, epistaxis due to blood heat, swelling, and pain of eye, corneal opacity and vision obstruction, liver depression and hypochondriac pain, dysmenorrhea, and traumatic injury. RPA is often used to treat irregular menstruation caused by blood deficiency or yin deficiency, spontaneous sweating and night sweating, contracture of the limbs, headaches, and dizziness caused by the ascendant hyperactivity of liver yang (Gao, 2012). The efficacy of these two traditional Chinese medicines places particular emphasis on different items. The differences in the properties of Chinese traditional medicines based on either RPR or RPA are outlined in **Table 2**.

**Table 2:** Differences between RPR and RPA.

Classification	RPR	RPA
Category	Heat-clearing blood-cooling medicinal	Blood-tonifying medicinal
Flavors and natures	Slightly cold, bitter	Slightly cold, bitter, and sour
Meridian entry	The liver	The liver and the spleen
Function	Clear heat and cool the blood, dissipate stasis to relieve pain	Nourish blood and regulate menstruation, arrest yin to check sweating, emolliate the liver and relieve pain, calm and repress liver yang
Indications	Heat entering construction-blood, macula, epistaxis, swelling, and pain of eye, corneal opacity and vision obstruction, liver depression and hypochondriac pain, dysmenorrhea, and traumatic injury	Irregular menstruation, spontaneous sweating and night sweating, contracture of the limbs, headache, and dizziness

Radix Paeoniae Rubra (RPR), Radix Paeoniae Alba (RPA).

### Differences in the Chemical Constituents of RPR and RPA

RPR and RPA are collectively referred to as PLP due to their close origins and similar chemical compositions (Yan et al., 2018). Modern medicinal chemistry and pharmacological studies have shown that the major biologically active ingredients of PLP include terpenoids, polyphenolic compounds, and volatile oils (Li et al., 2009; Xu et al., 2009; Yu et al., 2015; Liu et al., 2016). Due to the differences in growth environment and the processing methods after harvesting of these medicinal materials, the content ratio of these components in these two different medicinal herbs is often different. RPR is the dried root of *Paeonia lactiflora* Pall. or *Paeonia veltchii* Lynch. There are significant differences between the roots of these two species. Not only are the contents of certain constituents different, there are also peak-to-peak ratio differences, which represents a fingerprint-like pattern to delineate the source of the medicinal material. This can cause differences in efficacy and quality to some extent in traditional Chinese medicines derived from different sources of RPR (Xu et al., 2009; Parker et al., 2016).

Researchers using fast high-performance liquid chromatography (HPLC), combined with diode array detection (DAD) and electrospray ionization time-of-flight mass spectrometry (ESI-TOFMS) have been able to rapidly separate and sensitively identify the main components of RPR. A total of 26 components were screened and identified in RPR, including 11 monoterpene glycosides, 11 galloylglucoses, and 4 other phenolic compounds (Liu et al., 2009). Another group of researchers used advanced instruments such as high-speed, ultra-performance liquid chromatography (UPLC) and time-of-flight mass spectrometry (TOF-MS) to measure the chemical composition of RPA. A total of 40 components were simultaneously separated, including 29 monoterpene glycosides, 8 galloylglucoses, and 3 distinct phenolic compounds (Li et al., 2009).

The monoterpene glycosides of PLP mainly include paeoniflorin, albiflorin, oxypaeoniflorin, benzoyl paeoniflorin, and benzoyl hydroxy paeoniflorin. These chemical compounds are collectively referred to as total glucosides (Zou et al., 2003; He et al., 2013). The polyphenolic compounds in RPR are mainly composed of a variety of chemical components such as galloylglucoses and paeonol. They are also the main medicinal components of disaccharide medicinal materials that have been verified by modern pharmacology and are generally recognized by the pharmaceutical industry as having efficacy for the treatment of human pathologies.

There are also differences in the chemical composition of RPR and RPA from different origins. Researchers have measured the composition of multiple samples using liquid chromatography coupled with an ion trap and time-of-flight mass spectrometry (LC-IT-TOF-MS) (Shi et al., 2016) using a high-performance liquid chromatography (HPLC) fingerprint method (He et al., 2012; Zha et al., 2012).

RPR is mostly wild and does not need to be processed. In contrast, RPA is mostly cultivated (Wang et al., 2014a), and it takes a series of complicated processing procedures such as soaking, scraping, and cooking before it is used as a medicine. In addition, volatile components are lost extremely easily during processing, and chemical conversions may occur (Zhu et al., 2018). Studies have been carried out performing qualitative and quantitative tests on the main components of RPR and RPA. These results show that paeoniflorin and polyphenolic compounds (such as gallic acid, methyl gallate, galloyglucoses, and paeonol) are higher in RPR (*P* < 0.05 or *P* < 0.01). However, the monoterpene glycosides, in addition to paeoniflorin (including albiflorin), is significantly higher in RPA (*P* < 0.01), as they are the second main components after paeoniflorin (Liu et al., 2015; Yu et al., 2015).

**Figure 2:**
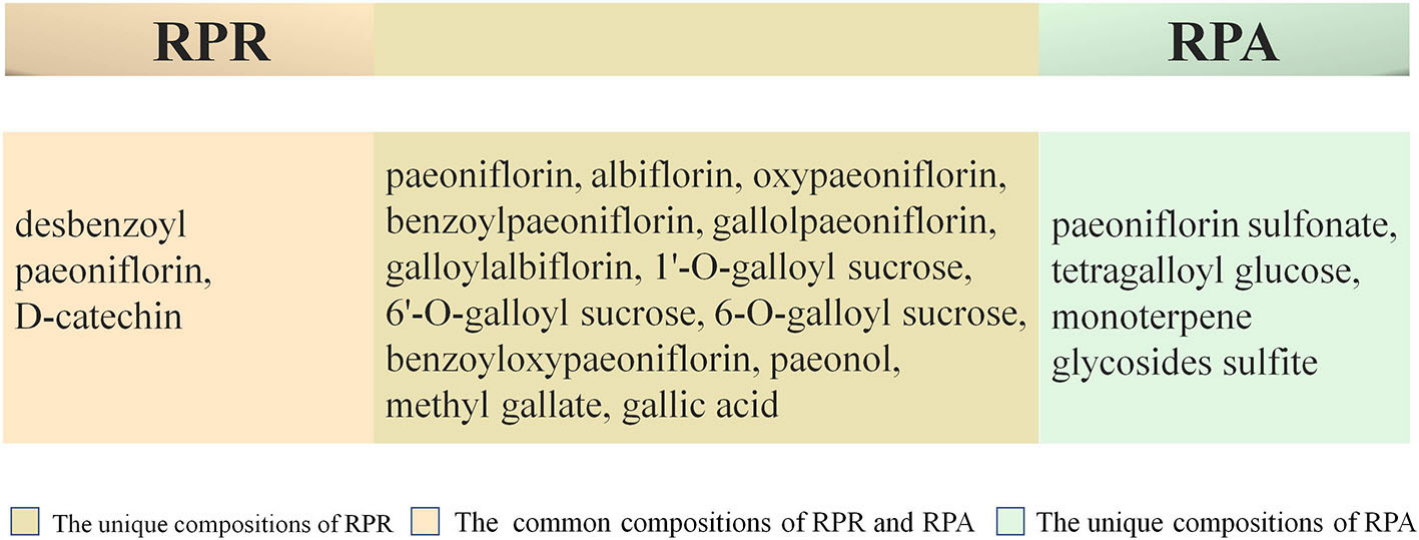
Main chemical compositions of RPR and RPA. *Radix Paeoniae Rubra* (RPR), *Radix Paeoniae Alba* (RPA).

**Figure 3:**
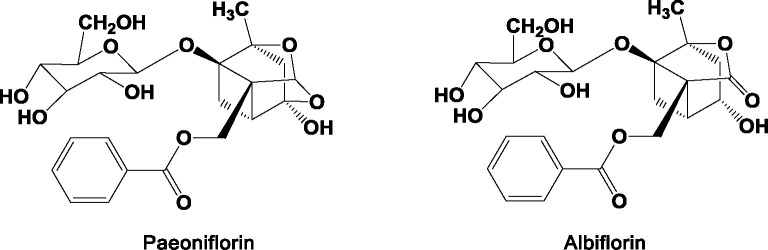
Chemical structures of paeoniflorin and albiflorin.

In addition, in order to make the appearance of RPA pieces white and beautiful, and also to prevent mildew and insects, some pharmacists adopt sulfur to fumigate RPA (Zhu et al., 2015). This process causes significant changes in the chemical composition of RPA pieces, as well as altering the pharmacokinetics (Kong et al., 2018) and changing the flavors and natures of the RPA. As the degree of sulfur smoked is increased, the content of paeoniflorin is subsequently reduced, and some of this is turned into paeoniflorin sulfonate. No paeoniflorin sulfonate, however, is detected within samples that have not been fumigated with sulfur. Paeoniflorin sulfonate is not a component originally found in RPA. This represents a novel strategy for tracking RPA by testing paeoniflorin sulfonate. In fact, sulfur fumigation alters the pharmacokinetics and reduces the safety and effectiveness of RPA. Sulfur fumigation is not recommended for post-harvest handling of RPA (Li et al., 2017). As far as the current state of chemical composition research is concerned, there is no recognized marker component that distinguishes between RPR and RPA. Main chemical compositions are presented in between RPR and RPA in **Figure 2** (Zhou et al., 2003; Liu et al., 2015; Yu et al., 2015; Shi et al., 2016). The chemical structures of paeoniflorin and albiflorin are presented in **Figure 3**. The differences in chemical compositions between RPR and RPA are highlighted in **Table 3** (Zhou et al., 2003; Liu et al., 2015; Yu et al., 2015).

**Table 3:** The differences in chemical compositions between RPR and RPA ($\bar{x}$ ± *s*, mg·g^−1^).

Classification	Paeoniflorin	Albiflorin	Gallic acid	Galloyglucoses	Methyl gallate	Paeoniflorin sulfonate
RPR	76.2 ± 1.11	2.15 ± 1.07	3.43 ± 0.77	21.51 ± 15.97	7.32 ± 6.01	ND
RPA	27.9 ± 1.01^b^	12.61 ± 3.09 ^b^	2.01 ± 0.36^b^	11.20 ± 2.27^a^	2.58 ± 1.06 ^b^	13.71 ± 8.78^b^

Radix Paeoniae Rubra (RPR), Radix Paeoniae Alba (RPA), N(RPR)=15, N(RPA)=17, ^a^P < 0.05, ^b^P < 0.01 compared with RPR. ND means no corresponding ingredients.

### The Pharmacological Actions of RPR and RPA

#### The Pharmacological Actions of RPR

Extensive accumulated evidence indicates that RPR has a variety of pharmacological actions, especially in terms of liver protection, and its effects on the heart and blood vessels.

**Figure 4:**
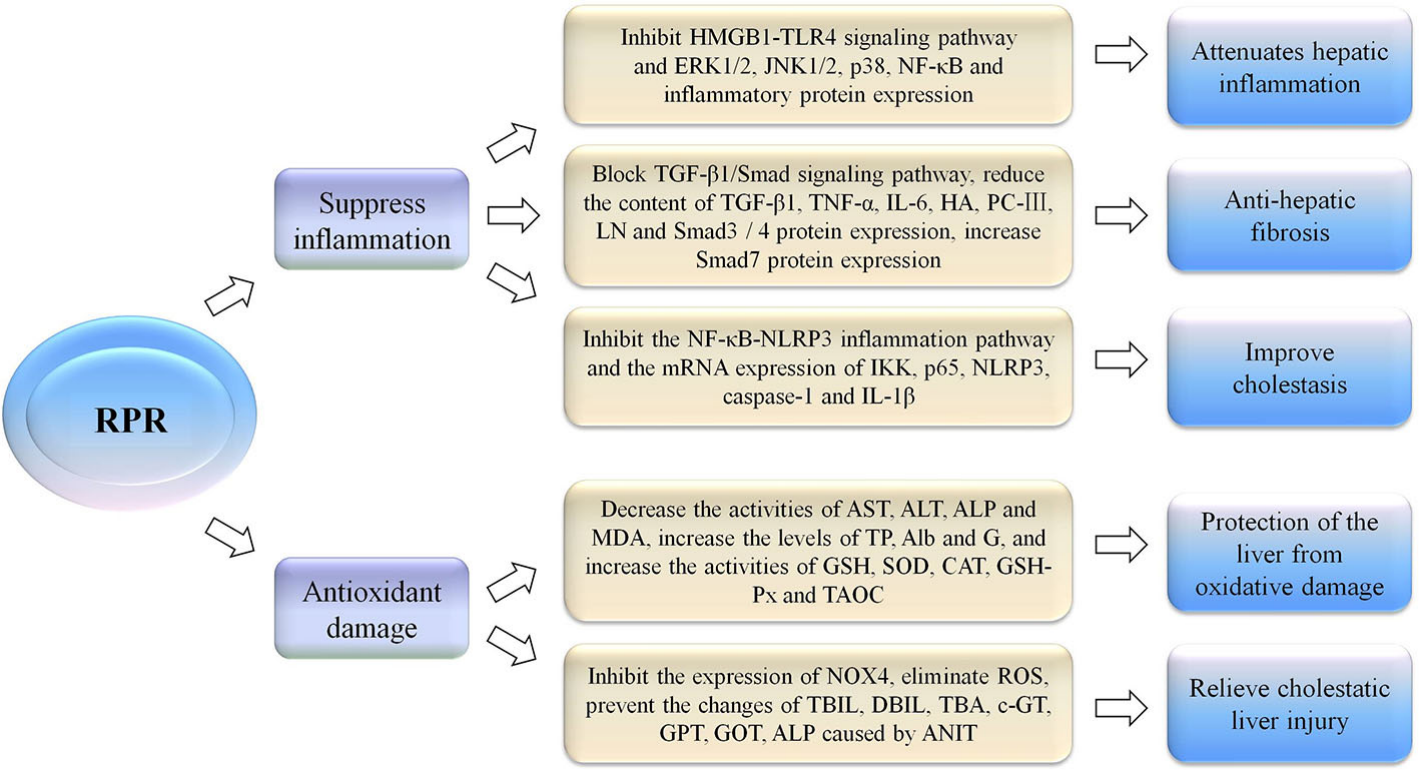
The hepatoprotective effect of RPR by inhibition of inflammatory reactions or resistance to oxidative damage.

At present, studies have shown that the possible mechanism of the hepatoprotective effect of RPR is related to the inhibition of inflammatory reactions or resistance to oxidative damage and free radical scavenging (**Figure 4**). There are three chemical components with prominent hepatoprotective activities in RPR, namely paeoniflorin, ethyl palmitate, and ethyl linoleate (Lu et al., 2012). Experiments have shown that paeoniflorin can inhibit ischemia/reperfusion (I/R)-induced hepatocyte apoptosis and cysteinyl aspartate specific proteinase 3 (caspase-3) activation, as well as dampening I/R-induced neutrophil infiltration and pro-inflammatory cytokine production. The protective effect on the liver is associated with the inhibition of the I/R-activated high mobility group box-1 (HMGB1)-toll-like receptors 4 (TLR4) signaling pathway to attenuate hepatic inflammatory response (Xie et al., 2018). In the CCl4-induced acute liver injury model, RPR water extracts protect the liver from CCl4-induced oxidative damage in rats, which was associated with antioxidant and free radical scavenging activity (Li et al., 2011; Wang et al., 2012). At the same time, studies have also shown that paeoniflorin, the main component of RPR, can regulate glutathione to protect against liver damage (Jiang et al., 2012; Zhao et al., 2013). In addition, research results have shown that RPR has anti-hepatic fibrosis efficacy, and its mechanism may be related to its blocking of the TGF-β1/Smad signaling pathway, providing novel mechanisms for the treatment of liver fibrosis (Gao et al., 2012). The total glucosides of RPR have a specific role in resolving jaundice that may be related to the activity of uridine diphosphoglucuronyl transferase (Luo et al., 2010). These results indicated that RPR can ameliorate alpha-naphthylisothiocyanate-induced cholestasis in rats. The mechanism of anti-inflammation may be related to regulating the nuclear factor kappa B (NF-κB)-NLR family pyrin domain containing 3 (NLRP3) inflammasome pathway (Ma et al., 2018).

*Radix Paeoniae Rubra* (RPR), high mobility group box-1 (HMGB1), toll-like receptors 4 (TLR4), extracelluar signal regulated kinase 1/2 (ERK 1/2), c-Jun N-terminal kinase 1/2 (JNK 1/2), p38 mitogen-activated protein kinase (p38), nuclear factor kappa B (NF-κB), transforming growth factor-β1 (TGF-β1), mothers against decapentaplegic protein (Smad), tumor necrosis factor-α (TNF-α), interleukin-6 (IL-6), hyaluronic acid (HA), procollagen type III (PC-III), Laminin (LN), NLR family pyrin domain containing 3 (NLRP3), inhibitor of nuclear factor kappa-B kinase (IKK), p65 is one of the members of the NF-κB family and is the most typical promoter in the nuclear transcription factor family, cysteinyl aspartate specific proteinase 1 (caspase-1), interleukin-1β (IL-1β), aspartate aminotransferase (AST), alanine aminotransferase (ALT), alkaline phosphatase (ALP), total protein (TP), albumin (Alb), globulin (G), malondialdehyde (MDA), glutathione (GSH), superoxide dismutase (SOD), catalase (CAT), glutathione peroxidase (GSH-Px), total antioxidant capacity (TAOC), NADPH oxidase 4 (NOX4), reactive oxygen species (ROS), total bilirubin (TBIL), direct bilirubin (DBIL), total bile acid (TBA), c-glutamyltranspeptidase (c-GT), glutamate-pyruvate transaminase (GPT), glutamate- oxaloacetic transaminase (GOT), alkaline phosphatase (ALP), 1-naphthyl isothiocyanate (ANIT).

**Figure 5:**
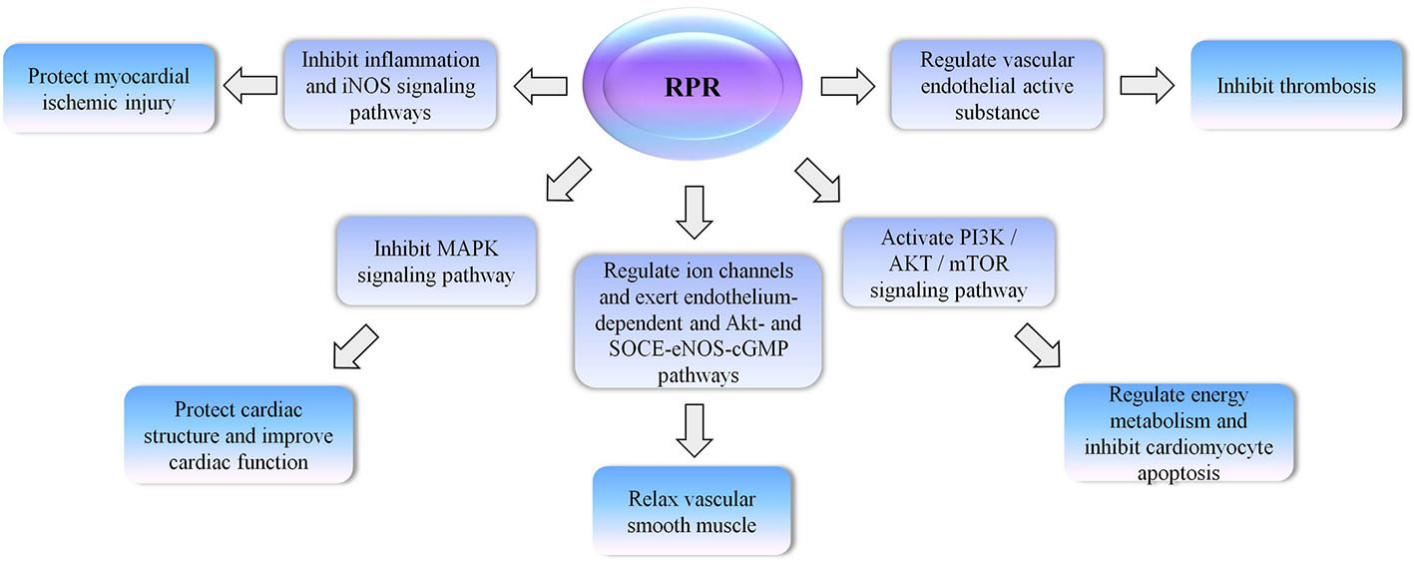
The therapeutic effect of RPR on cardiovascular disease.

The therapeutic effect of RPR on cardiovascular disease is also very significant (**Figure 5**). In an acute myocardial infarction (AMI) rat model, RPR extract plays a positive role in regulating cardiac enzymes, cytokines, oxidative stress, coagulation, and apoptosis (Mo et al., 2011). It was previously discovered that inducible nitric oxide synthase (iNOS) is a catalytic enzyme involved in the synthesis of nitric oxide (NO). NO plays an important regulatory role in the cardiovascular, immune, and nervous systems. These results indicated that paeoniflorin may improve myocardial infarction by inhibiting the inflammation and iNOS signaling pathways (Chen et al., 2015). Experimental research shows that paeoniflorin relieves heart hypertrophy, heart fibrosis and inflammation, and improves left ventricular function. Moreover, paeoniflorin decreases blood pressure and increases hemodynamic indices. In conclusion, the protective effect of paeoniflorin on cardiac remodeling is related to the inhibition of mitogen-activated protein kinase (MAPK) signaling pathway (Liu et al., 2019). An ethanol extract of RPR relaxes vascular smooth muscle by the endothelium-dependent and protein kinase B (AKT) and store-operated calcium entry (SOCE)-endothelial nitric oxide synthase (eNOS)-cyclic guanosinc monophosphate (cGMP)-mediated pathways by activating K(Ca) and K(ATP) channels and inhibiting L-type Ca^2+^ channels (Jin et al., 2012). Studies have shown that terpene glucoside protects the heart from ISO-induced myocardial ischemia by activating the phosphatidy linositol 3-kinase (PI3K)/AKT/mammalian target of rapamycin (mTOR) signaling pathway to improve cardiac energy metabolism and inhibit cardiomyocyte apoptosis. The effect of improving energy metabolism in the body and alleviating myocardial damage is related to terpene glucosides. As the main components of RPR, terpene glucosides improve energy metabolism in the body and reduce myocardial damage. The expression of p-AKT and p-mTOR are also significantly increased, while the levels of caspase-3 and Bax/Bcl-2 (Bcl-2 associated x protein/B-cell lymphoma-2) are significantly decreased (Ke et al., 2017). RPR has an inhibitory effect on thrombosis, the compounds of which can regulate vascular endothelial active substances and activate blood flow and anticoagulation programs (Xie et al., 2017).

*Radix Paeoniae Rubra* (RPR), inductible nitric oxide synthase (iNOS), mitogen-activated protein kinase (MAPK), protein kinase B (AKT), store-operated calcium entry (SOCE), endothelial nitric oxide synthase (eNOS), cyclic guanosinc monophosphate (cGMP), phosphatidy linositol 3-kinase (PI3K), mammalian target of rapamycin (mTOR).

In summary, RPR can improve microcirculation, dilate blood vessels, prevent myocardial ischemia and prevent thrombosis. These effects are consistent with the efficacy of traditional Chinese medicine for activating blood and resolving stasis.

#### The Pharmacological Actions of RPA

RPA also displays a variety of pharmacological efficacies, which have a considerable impact on the nervous and immune systems.

The therapeutic effects of RPA on the nervous system can be summarized as follows: neuroprotective, antidepressant, sedative, and analgesic and anticonvulsant. Total glucosides of paeony can exhibit pre-protection against neurotoxicity by reducing the absorption of toxic alkaloids by the brain (Hou et al., 2017; Li et al., 2018). Albiflorin is the main ingredient of RPA, and it can lower the levels of neuronal nitric oxide synthase (nNOS), and the antinociceptive activity is at least partially related to calcium channels (Zhang et al., 2016b). Paeoniflorin is less abundant in RPA, as the main active component for neuroprotection, and the protective effect on the nervous system may be achieved by regulating the Ca^2+^/calmodulin-dependent protein kinase II (CaMKII)/cAMP-response element binding protein (CREB) signaling pathway (Zhang et al., 2017). Experimental data indicate that albiflorin may be a potential anti-depressant drug. Albiflorin inhibits the uptake of 5-hydroxytryptamine (5-HT) and norepinephrine (NE) and exhibits strong binding affinities for two neurotransmitter transporters. The hippocampal brain-derived neurotrophic factor (BDNF) expression levels and the hippocampal 5-HT, 5-hydroxyindole acetic acid (5-HIAA) and noradrenaline (NA) levels are all up-regulated after RPR treatment (Jin et al., 2016; Wang et al., 2016a). Professor Li carried out a series of animal experiments to observe the analgesic and sedative effects of different processed products of RPA, including the warm bath shrink mouse tail method, forced swimming tests in mouse, and the mouse ear swelling anti-inflammatory method. The results showed that different processed products of RPA all had analgesic, sedative, and anti-inflammatory actions. RPA processed by wine sautéing and RPA processed by vinegar sautéing were better than RPA without processing and RPA processed by pure sautéing (Li et al., 2017). It has been reported also that total glucosides of paeony have anticonvulsant effects (Zhang et al., 1994). These effects are similar to the efficacy of traditional Chinese medicine for relaxing tension and relieving pain.

Total glucosides of paeony (TGP), in capsule form, are widely used in the treatment of autoimmune diseases, such as rheumatoid arthritis.

**Figure 6:**
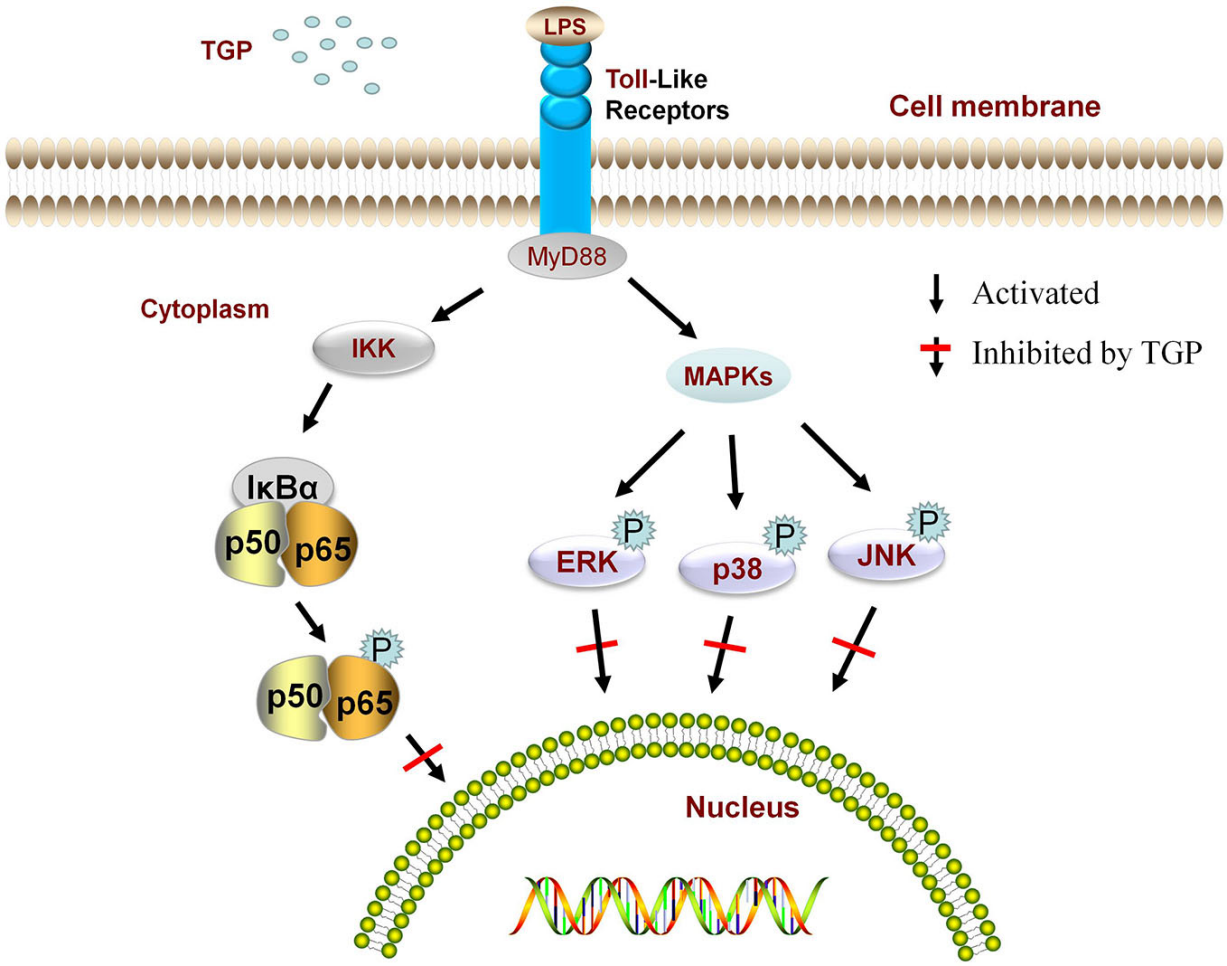
Regulation of the MAPK and NF-κB signaling pathways by RPA.

Dendritic cells play a key role in the initial stage of immunization. TGP can selectively block the activation of TLR4/5 to inhibit the maturation of dendritic cells, and thus reduce immune-mediated responses *in vivo* (Zhou et al., 2012). The MAPK signaling pathway plays a crucial role in the inflammatory process. TGP inhibits experimental autoimmune uveitis-induced p38 mitogen-activated protein kinase (p38), extracellular signal regulated kinase (ERK) and c-Jun N-terminal kinase (JNK) phosphorylation, and TGP mediates mouse autoimmune uveitis by inhibiting the MAPK signaling pathway (Huang et al., 2018). This study found that TGP treatment could reduce phosphorylation of IκBα and NF-κB p65 proteins, thereby reducing nuclear translocation of NF-κB p65 in HaCaT cells (Wang et al., 2016b). **Figure 6** shows the regulation of the MAPK and NF-κB signaling pathways by RPA.

Total glucosides of paeony (TGP), Lipopolysaccharide (LPS), myeloid differentiation factor 88 (MyD88), phosphorylation (P), inhibitor of nuclear factor kappa-B kinase (IKK), mitogen-activated protein kinases (MAPKs), nuclear transcription factorκB (NF-κB), IκBα is an important member of the IκB (inhibitor of NF-κB) family, NF-κB p50 and p65 are the most representative dimer complexes in the nuclear transcription family, p65 is one of the members of the NF-κB family and is the most typical promoter in the nuclear transcription factor family. extracellular signal regulated kinase (ERK), c-Jun N-terminal kinase (JNK), p38 mitogen-activated protein kinase (p38).

Studies have shown that some kinds of autoimmune diseases, such as primary Sjogren’s syndrome and inflammatory bowel disease, can be treated by regulating regulatory T cells/T helper cell 17 cells (Chen et al., 2019; Li et al., 2019).

**Figure 7:**
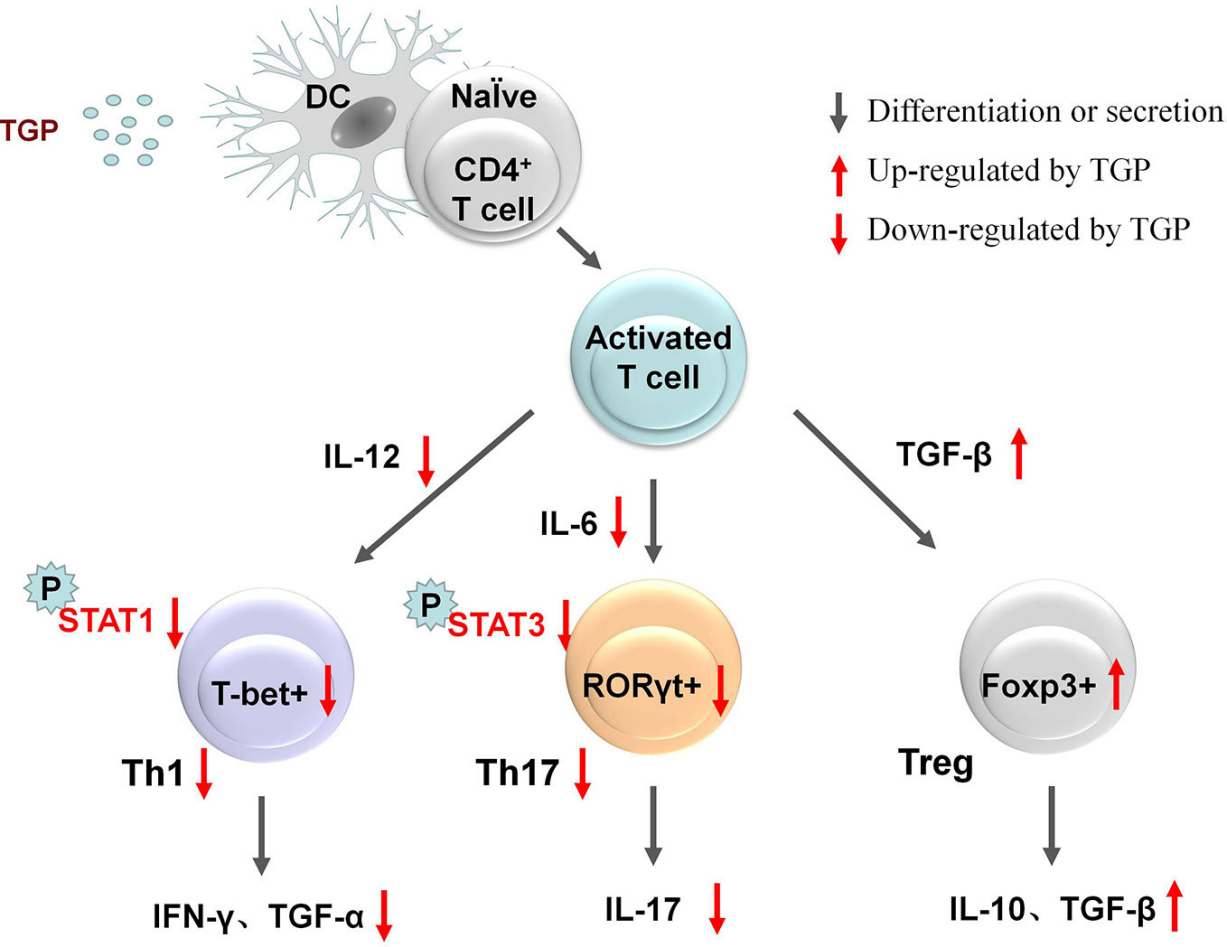
Regulation of the immune system by TGP.

TGP treatment can increase the expression of TGF-β and IL-10, up-regulate peripheral Treg cells and the transcription factor Foxp3 in Treg cells (Zhao et al., 2012; Zhao et al., 2018) and inhibit the levels of Th17-related cytokines like IL-6 and IL-17 (Lin et al., 2012; Lin et al., 2017). IL-1β is also down-regulated by TGP treatment (Zhang et al., 2014). TGP can down-regulate the expression of p-STAT3 by up-regulating miR-124 as well (Zhao et al., 2018). Studies have shown that signal transducer and activator of transcription 1 (STAT1) and STAT3 phosphorylation are inhibited by TGP. Retinoid-related orphan nuclear receptor gamma t (RORγt) is a specific transcription factor that promotes Th17 differentiation, and T-bet is selectively expressed in Th1, both of which are down-regulated by TGP exposure (Lin et al., 2012; Wang et al., 2014b). TGP can selectively down-regulate the production and secretion of peripheral Th1 cells (Wang et al., 2014b) and Th1 type cytokines such as transforming growth factor α (TGF-α) (Zhao et al., 2018), interleukin 12 (IL-12) and interferon-γ (IFN-γ) (Li and Jiang, 2019). **Figure 7** outlines the changes in cytokines during immunization.

Total glucosides of paeony (TGP), dendritic cell (DC), signal transducer, and activator of transcription 1 (STAT1), signal transducer, and activator of transcription 3 (STAT3), T-bet+ is a newly identified Th1 specific T-box transcription factor selectively expressed in Th1 cells, retinoid-related orphan nuclear receptor gamma t (RORγt), forkhead box p3 (foxp3), regulatory cell (Treg), interferon-γ (IFN-γ), helper T cell (Th), interleukin (IL), transforming growth factor α (TGF-α), transforming growth factor β (TGF-β), phosphorylation (P).

### Common Pharmacological Actions

Because they contain roughly the same compositions (**Figure 2**), RPR and RPA have many similar efficacies, the typical of which are anti-inflammatory and anti-tumor effects.

The chemical components with anti-inflammatory action are mainly paeoniflorin and paeonol, which are common compositions of RPR and RPA. Paeoniflorin down-regulates matrixmetallo proteinase-2 (MMP-2), matrixmetallo proteinase-9 (MMP-9), iNOS, and cyclooxygenase-2 (COX-2) levels (Ni et al., 2016) and inhibits the MAPK/NF-κB signaling pathways and apoptosis (Gu et al., 2017). Paeoniflorin also promotes the upregulation of pro-inflammatory mediators such as tumor necrosis factor-α (TNF-α), interleukin 1β (IL-1β), iNOS, COX-2, and 5-lipoxygenase (5-LOX), and the activations of JNK and p38 MAPK, so as to protect from ischemic brain damage (Guo et al., 2012). Paeonol can reverse the overproduction of iNOS, COX-2, matrixmetallo proteinase-1 (MMP-1), matrixmetallo proteinase-3 (MMP-3), and matrixmetallo proteinase-13 (MMP-13), and inhibit NF-κB activation, as well as PI3K and AKT phosphorylation (Lou et al., 2017).

Studies have shown that the main components with anti-tumor activity in RPR and RPA are paeoniflorin (Yang et al., 2018), paeonol (Saahene et al., 2018), gallic acid (Lin et al., 2016) and methyl gallate (Lee et al., 2010). Researchers have used the extracts with paeoniflorin, gallic acid, and methyl gallate on mice with bladder tumors. The anti-proliferative effects of the extract reduced certain cell cycle populations, mainly G1 phase cells, and caused a significant sub-G population elevation (Lin et al., 2016). In experiments on mice carrying DU145 tumor cells, paeonol promoted apoptosis of DU145 cells, enhanced the activity of caspase-3, caspase-8, and caspase-9, decreased the expression of Bcl-2 and increased the expression of Bax. The phosphorylation of AKT and mTOR was also reduced, and the inhibition of proliferation of DU145 by paeonol and PI3K/AKT inhibitors were synergistic (Xu et al., 2018).

### Comparison of the Effects of RPR and RPA on Improving Microcirculation

Both RPR and RPA can improve hemorheological abnormalities and protect vascular endothelial function, but RPR is superior to RPA in improving microcirculation (**Table 4**) (Wang et al., 2010; Zhang et al., 2016a; Jia et al., 2018). The inhibitory effect of 80% ethanol total extract of RPR on platelet aggregation was significantly better than that of total extract of RPA by adenosine diphosphate-induced platelet aggregation in rats (Wang et al., 2010). By observing the improvement of hemorheology of acute blood stasis model rats by RPR and RPA, it was found that RPR can significantly improve the whole blood viscosity of blood stasis model rats at different shear rates, prolong activated partial thromboplastin time (APTT), prothrombin time (PT), thrombin time (TT), reduce platelet adhesion rate, reduce the amount of thromboxane B2 (TXB2) in serum, increase the amount of 6-keto-prostaglandin 1α (6-keto-PGF1α), and up-regulate the expression levels of platelet eNOS and p-eNOS protein. RPA had no significant effect on the whole blood viscosity of rats with blood stasis. However, it can significantly prolong APTT and TT, reduce platelet adhesion rate, and increase the amount of 6-keto-PGF1α in serum (Zhang et al., 2016a). By observing the relaxant effect of RPR and RPA extracts on superior mesenteric artery of rats given different precontraction stimuli, it was found that RPR and RPA extracts had no relaxant effect on basal state vessels on vessels with intact endothelium. On endothelium-intact and endothelium-denuded vessels precontracted by different substances, the extract of RPR had a significant relaxant effect on precontracted vessels with K^+^ (60 mM), phenylephrine (PE, 10^−5^ M) and 5-HT (10^−5^ M) within the dosage range of 10^−4^ to 10^−3^ g·L^−1^, while the relaxant effect of RPA extract was not significant (Jia et al., 2018). Both RPR and RPA could reduce the blood viscosity of rats with acute blood stasis syndrome, paeoniflorin was the common active component of the two, albiflorin had less effect on blood stasis syndrome than paeoniflorin. The effect of RPR on blood stasis syndrome was better than that of RPA, which was related to its more content of paeoniflorin, and paeoniflorin had a stronger effect on improving hemorheological abnormalities and protecting vascular endothelial function (Le et al., 2019).

**Table 4:** Effect of RPR and RPA on improving microcirculation.

	RPR	RPA
Maximum platelet aggregation rate/%	31.87 ± 6.13	44.70 ± 3.70^b^
Whole blood viscosity/(mPa·s) (200 s^−1^)	3.99 ± 0.37	4.53 ± 0.17^b^
Whole blood viscosity/(mPa·s) (30 s^−1^)	5.21 ± 0.29	6.13 ± 0.64^b^
Whole blood viscosity/(mPa·s) (3 s^−1^)	10.42 ± 2.70	11.80 ± 2.80^a^
APTT/s	26.47 ± 1.15	22.80 ± 1.85^a^
PT/s	19.13 ± 1.15	15.57 ± 1.35^a^
TT/s	29.73 ± 3.31	23.67 ± 2.57^a^
Vasodilation rate (K^+^,60 mM)/%	51.23 ± 10.45	2.46 ± 1.81^b^
Vasodilation rate (PE,10^−5^ M)/%	61.44 ± 13.48	3.70 ± 1.28^b^
Vasodilation rate (5-HT, 10^−5^ M)/%	76.24 ± 23.88	3.89 ± 1.64^b^

Radix Paeoniae Rubra (RPR), Radix Paeoniae Alba (RPA), activated partial thromboplastin time (APTT), prothrombin time (PT), thrombin time (TT), Phenylephrine (PE), 5-hydroxytryptamine (5-HT), ^a^P < 0.05, ^b^P < 0.01 compared with RPR.

## Discussion

The sources and compositions of RPR and RPA are highly similar. Owing to the differences in distribution areas and processing methods, the effects of these two compounds are different. According to the Chinese Pharmacopoeia, the identification of the two is determined by measuring the content of paeoniflorin. However, results show that there is a bias in identification using paeoniflorin only (Zhou et al., 2003). Albiflorin, as a specific component of RPA, has very little content in RPR and can be used as a new identification index. At present, the research on these two is mostly based on their active ingredients, such as paeoniflorin, albiflorin, and paeonol. It is difficult to directly compare the differences in their efficacy. Traditional Chinese medicine has the characteristics of using multi-component, multi-target, and multi-effect agents. Studies on the pharmacological mechanism of single components or portions of compounds need to be further conducted, as the pharmacological actions of other components have yet to be confirmed. In conclusion, RPR displays outstanding hepatoprotective and cardiovascular effects, RPA has a therapeutic effect on the nervous and immune systems, and both of them have anti-inflammatory and anti-tumor pharmacological effects, which provides a broad platform for the development of new drugs in the future.

## Author Contributions

Y-QT wrote the first draft of the manuscript. H-WC and JL conceived and revised the manuscript. Q-JW revised sections of manuscript. H-WC and Y-QT drew article figures and tables. All authors contributed to the article and approved the submitted version.

## Funding

This study was supported by the National Natural Science Foundation of China (No. 81973836), the China Association for Science and Technology Youth Talent Project (No.2017QNRC001), and the China Academy of Chinese Medical Sciences—Traditional Chinese Medicine Science and Technology Achievement Leading Project (No. ZZ13-ZD-04).

## Conflict of Interest

The authors declare that the research was conducted in the absence of any commercial or financial relationships that could be construed as a potential conflict of interest.
